# The Inhibition of Lipase and Glucosidase Activities by Acacia Polyphenol

**DOI:** 10.1093/ecam/neq043

**Published:** 2011-02-14

**Authors:** Nobutomo Ikarashi, Rumi Takeda, Kiyomi Ito, Wataru Ochiai, Kiyoshi Sugiyama

**Affiliations:** Department of Clinical Pharmacokinetics, Hoshi University, Tokyo 142-8501, Japan

## Abstract

Acacia polyphenol (AP) extracted from the bark of the black wattle tree (*Acacia mearnsii*) is rich in unique catechin-like flavan-3-ols, such as robinetinidol and fisetinidol. In an *in vitro* study, we measured the inhibitory activity of AP on lipase and glucosidase. In addition, we evaluated the effects of AP on absorption of orally administered olive oil, glucose, maltose, sucrose and starch solution in mice. We found that AP concentration-dependently inhibited the activity of lipase, maltase and sucrase with an IC_50_ of 0.95, 0.22 and 0.60 mg ml^−1^, respectively. In ICR mice, olive oil was administered orally immediately after oral administration of AP solution, and plasma triglyceride concentration was measured. We found that AP significantly inhibited the rise in plasma triglyceride concentration after olive oil loading. AP also significantly inhibited the rise in plasma glucose concentration after maltose and sucrose loading, and this effect was more potent against maltose. AP also inhibited the rise in plasma glucose concentration after glucose loading and slightly inhibited it after starch loading. Our results suggest that AP inhibits lipase and glucosidase activities, which leads to a reduction in the intestinal absorption of lipids and carbohydrates.

## 1. Introduction

Acacia is an evergreen tree belonging to the genus *Acacia* in the legume family that is widely found throughout the Australian and African continents. The extract of *Acacia catechu* duramen is called gambir and has long been used as an astringent and antibacterial to treat stomatitis and diarrhea in Japan and China. Studies have also reported that the powdered seeds of *A. catechu* and *A. melanoxylon* exhibit hypoglycemic actions by increasing insulin secretion in nondiabetic rats and rabbits [1, 2]. In Europe, acacia polyphenol (AP) extracted from the bark of the black wattle tree (*A. mearnsii*) has been used to eliminate wine sediment. Australian aborigines also eat the young leaves and beans of *A. mollissima*.

We previously conducted a study using KKAy mice, a model for obesity and type 2 diabetes, to evaluate the anti-obesity and anti-diabetic effects of AP [3]. We found that AP significantly inhibited body weight gain and improved diabetes and insulin resistance. The anti-obesity actions of AP appear to be attributable to increased expression of energy expenditure-related genes in skeletal muscle and liver and to decreased fatty acid synthesis and fat intake in the liver. AP also reduces hyperglycemia and hyperinsulinemia by increasing adiponectin secretion and suppressing tumor necrosis factor-alpha secretion by white adipocytes, and elevating the expression of GLUT4 in skeletal muscle, in addition to reducing obesity.

AP contains about 80% polyphenols with a molecular weight (MW) of 300–3000 kDa [4, 5]. In general, polyphenol constituents with a low MW can easily be absorbed, compared to higher MW constituents. Low-MW AP constituents are thought to be absorbed in the intestine, thereby altering gene expression in KKAy mice while high-MW AP constituents, which are not thought to be absorbed into the body, most likely exhibit inhibitory effects against obesity and diabetes in the gastrointestinal tract. To date, various natural products containing polyphenols have been reported to have anti-obesity and anti-diabetic effects by inhibiting lipase activity and *α*-glucosidase activity [6–8]. Therefore, to elucidate the action of AP in the intestine, we conducted *in vitro* lipase activity inhibition and *α*-glucosidase activity inhibition tests. We also performed *in vivo* lipid tolerance and carbohydrate tolerance tests in order to evaluate the effects of AP on lipid and carbohydrate absorption.

## 2. Methods

### 2.1. Hot Water Extract of Acacia Bark

AP was donated by Mimozax Co., Ltd. (Hiroshima, Japan) and prepared according to the methods reported by Cutting [9]. Briefly, the powdered bark of South African *A. mearnsii* was pulverized and extracted for 30 min in a 10-fold volume of hot water (100°C), and then dried using a spray drier. The polyphenol content of the product, as determined by the Stiasny reaction [10–12], was 83.3%. The number-average MW (Mn) and weight-average MW (Mw) of AP were 812 and 1280, respectively, and robinetinidol and fisetinidol were the major constituents.

### 2.2. Materials

Orlistat, pancreatic lipase from porcine pancreas, maltose, sucrose, glucose, starch, maleic acid and rat intestinal acetone powder were purchased from Sigma-Aldrich Corp. (St. Louis, MO, USA). Triolein, lecithin, taurocholic acid, bathocuproine, 3-(2)-*tert*-butyl-4-hydroxyanisol and acarbose were purchased from Wako Pure Chemical Industries (Osaka, Japan). All other reagents were commercially available products of the highest available grade.

### 2.3. Animals

Male ICR mice (4 weeks old) were purchased from Sankyo Labo Service Corp., Inc. (Tokyo, Japan). Each mouse was kept at room temperature (22 ± 1°C) and 55 ± 10% humidity with a 12-h light/dark cycle (artificial illumination: 08:00–20:00 h) and acclimatized for 1 week before experiments. The present study was conducted in accordance with the Guiding Principles for the Care and Use of Laboratory Animals, as adopted by the Committee on Animal Research of Hoshi University.

### 2.4. Assay of Pancreatic Lipase Activity In Vitro

Lipase activity was determined by measuring the rate of release of oleic acid from triolein [13]. A suspension of 90 *μ*mol triolein, 12.6 *μ*mol lecithin and 9.45 *μ*mol taurocholic acid in 9 ml of 0.1 M Tris buffer (pH 7.0) was sonicated for 10 min for substrate dissolution. AP (final concentration: 0, 0.02, 0.1, 0.25, 0.5, 1.0 or 2.0 mg ml^−1^) and orlistat (final concentration: 0, 0.01, 0.1, 1, 10, 100 or 200 *μ*g ml^−1^), which was used as a positive control, were diluted with 0.1 M Tris buffer (pH 7.0). Next, 50 *μ*l of pancreatic lipase (2 mg ml^−1^ in Tris buffer), 100 *μ*l of substrate solution and 100 *μ*l of sample solution were mixed and incubation was carried out at 37°C for 30 min. The mixture was mixed with 1 ml of chloroform-*n*-heptane (1 : 1) containing 2% methanol and extracted by shaking for 5 min. The mixture was centrifuged at 1000 g for 5 min, and the upper aqueous phase was removed by suction. Copper reagent (700 *μ*l) was then added to the lower organic phase. The tube was then shaken for 5 min and the mixture was centrifuged at 1000 g for 5 min, after which 0.5 ml of the upper organic phase (which contained the copper salts of the extracted fatty acid) was treated with 0.5 ml of 0.1% bathocuproine in chloroform containing 0.05% 3-(2)-*tert*-butyl-4-hydroxyanisol, and its absorbance was measured at 490 nm using a U-2800 spectrophotometer (Hitachi High Technologies, Tokyo, Japan).

### 2.5. Assay of *α*-Glucosidase Activity In Vitro

*α*-Glucosidase activity was calculated by measuring the amount of glucose hydrolyzed from maltose or sucrose [14, 15]. Enzyme solutions were prepared using rat intestinal acetone powder as a source of *α*-glucosidase. One gram of rat intestinal acetone powder was homogenized with 9 ml of 0.1 M maleate buffer (pH 6.9), followed by centrifugation at 1000 g for 10 min at 4°C. The supernatant was diluted with 0.1 M maleate buffer by 20 times or two times, and was used as the enzyme solution for the maltase or sucrase reactions, respectively. AP (final concentration: 0, 0.02, 0.1, 0.25, 0.5, 1.0 or 2.0 mg ml^−1^) and acarbose (final concentration: 0, 0.01, 0.1, 1, 10 or 100 *μ*g ml^−1^), which was used as a positive control, were diluted with 0.1 M maleate buffer. Next, 200 *μ*l of sample solution and 200 *μ*l of maltose substrate solution (2% w/v in maleate buffer) or sucrose substrate solution (2% w/v in maleate buffer) was mixed, and pre-incubated at 37°C for 5 min. The enzyme reaction was initiated by adding 200 *μ*l of the enzyme solution. The enzyme reaction was allowed to proceed at 37°C for 60 min, and was then stopped by heating at 100°C for 10 min. The reaction mixture was kept on ice, and the mixture was centrifuged at 1000 g  for 10 min. Glucose in the supernatant was measured using a commercial assay kit (Glucose CII-Test Wako, Wako Pure Chemical Industries).

### 2.6. Oral Olive Oil Tolerance Test in Mice

For the lipid absorption test, mice were deprived of food for 18 h before the experiment. An AP solution was orally administered at 0.25, 0.5 or 1.0 g kg^−1^ body weight. Olive oil or distilled water was subsequently administered orally at 5 ml kg^−1^ body weight. Before and at 1.5, 3, 4.5 and 6 h after this administration, 60-*μ*l blood samples were taken from the eyeground. All samples were collected with a heparin-coated capillary tube and centrifuged (1000 g for 5 min) to separate the plasma. Plasma samples were frozen at –80°C until assay. Plasma triglyceride concentrations were measured with a Triglyceride E-test Wako (Wako Pure Chemical Industries).

### 2.7. Oral Carbohydrate Tolerance Test in Mice

For carbohydrate absorption tests, mice were deprived of food for 18 h before the experiment. An AP solution was orally administered at 0.25, 0.5 or 1.0 g kg^−1^ body weight. Glucose (2 g kg^−1^), maltose (2 g kg^−1^), sucrose (4 g kg^−1^) or starch (4 g kg^−1^) solution, or distilled water, was subsequently administered orally. Before and at 0.5, 1, 1.5, 2 and 3 h after administration, 60-*μ*l blood samples were taken from the eyeground. All samples were collected in heparin-coated capillaries and centrifuged (1000 g for 5 min) to separate the plasma. Plasma samples were frozen at –80°C until assay. Plasma glucose concentrations were then measured with a Glucose CII-Test Wako.

### 2.8. Statistical Analysis

All IC_50_ values were calculated from the corresponding dose inhibition curve. To determine total triglyceride and glucose absorption after lipid or carbohydrate administration, the triglyceride area under the plasma concentration-time curve (AUC) up to 6 h after olive oil administration and the glucose AUC up to 3 h after carbohydrate solution administration were determined by the trapezoidal method. ΔAUC was calculated by subtracting the AUC values from those in the no-olive oil or no-carbohydrate solution groups. Numerical data are expressed as means ± SD. Significance of differences was examined by analysis of variance, followed by Dunnett's test. Results with *P* < .05 were considered to be significant.

## 3. Results

### 3.1. Inhibitory Effects of AP on Lipase

**Figure 1:**
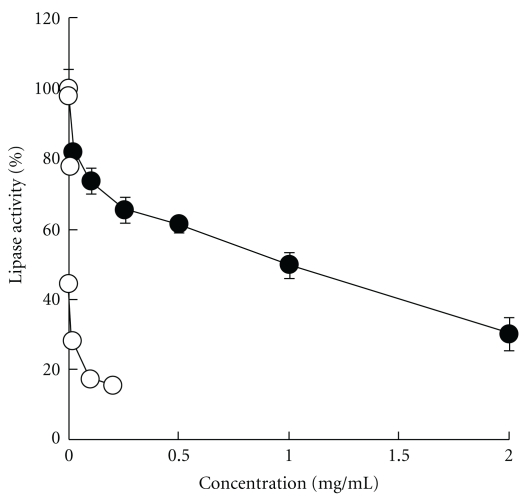
Inhibitory effects of AP (filled circle) or orlistat (open circle) on pancreatic lipase.The inhibitory activity of AP or orlistat on pancreatic lipase was measured as described in Methods section. Data represent means ± SD (*n* = 4).

Figure 1 shows the inhibitory effects of AP and orlistat on lipase. Orlistat, a lipase inhibitor used as the positive control, strongly inhibited lipase activity with an IC_50_ of 0.85 *μ*g ml^−1^. AP also concentration-dependently inhibited lipase activity with an IC_50_ of 0.95 mg ml^−1^.

### 3.2. Inhibitory Effects of AP on *α*-Glucosidase

**Figure 2:**
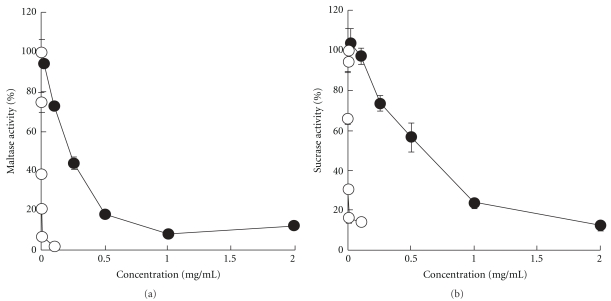
Inhibitory effects of AP (filled circle) or acarbose (open circle) on maltase (a) or sucrase (b). The inhibitory activity of AP or acarbose on maltase or sucrase was measured as described in ‘Methods' section. Data represent means ± SD (*n* = 4).

Figure 2 shows the inhibitory effects of AP and acarbose on maltase and sucrase. Acarbose, an *α*-glucosidase inhibitor used as the positive control, strongly inhibited maltase and sucrase activity with an IC_50_ of 0.071 and 0.49 *μ*g ml^−1^, respectively. AP also concentration-dependently inhibited maltase and sucrase activity with an IC_50_ of 0.22 and 0.60 mg ml^−1^, respectively.

### 3.3. Oral Olive Oil Tolerance Test in Mice

**Figure 3:**
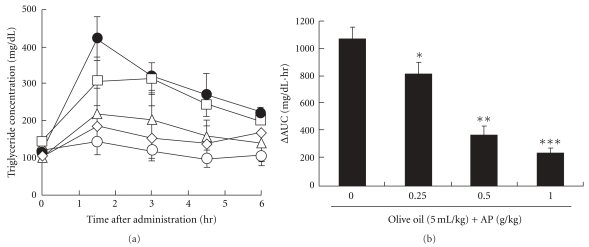
Olive oil tolerance test in mice. Blood triglyceride concentration profiles at 0 and 6 h after oral administration of AP and olive oil (a) and ΔAUC values of blood triglyceride at 6 h following oral administration of AP (0–1.0 g kg^−1^) and immediate oral administration of 5 ml/kg olive oil (b). ΔAUC was calculated by subtracting the AUC value from that in the no-olive oil group. open circle: no olive oil group filled circle: olive oil alone group, open square: olive oil/AP 0.25 g kg^−1^ group, open triangle: olive oil/AP 0.5 g kg^−1^ group, open diamond: olive oil/AP 1.0 g kg^−1^ group. Data represent means ± SD (*n* = 8). Dunnett's test: *P < .05, **P < .01 and ***P < .001 versus no-olive oil group.

Figure 3(a) shows the plasma triglyceride concentration profiles after oral administration of AP solution and olive oil to mice.  Figure 3(b) depicts the plasma triglyceride ΔAUC up to 6 h post-administration. For olive oil alone administration, plasma triglyceride concentrations were highest at 90 min post-administration, followed by a gradual decline. AP inhibited the rise in plasma triglyceride concentrations. Upon administration of 0.5 g kg^−1^ AP, triglyceride ΔAUC decreased to about 40% that in the olive oil alone group.

### 3.4. Oral Carbohydrate Tolerance Test in Mice

**Figure 4:**
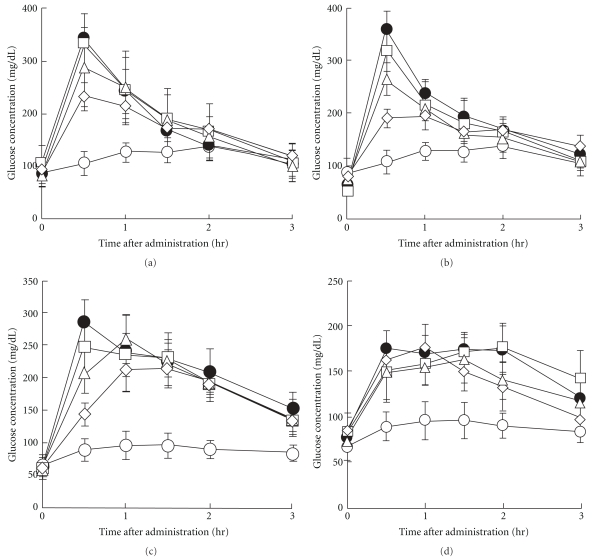
Carbohydrate tolerance test in mice. Blood glucose concentration profiles at 0–3 h after oral administration of AP (0–1.0 g kg^−1^) followed immediately by oral administration of glucose (2 g kg^−1^, a), maltose (2 g kg^−1^, b), sucrose (4 g kg^−1^, c) or starch (4 g kg^−1^, d) solution. Open circle: No carbohydrate group, Filled circle: carbohydrate alone group, open square: carbohydrate/AP 0.25 g kg^−1^ group, open triangle: carbohydrate/AP 0.5 g kg^−1^ group, open diamond: carbohydrate/AP 1.0 g kg^−1^ group. Data represent means ± SD (*n* = 8).

**Figure 5:**
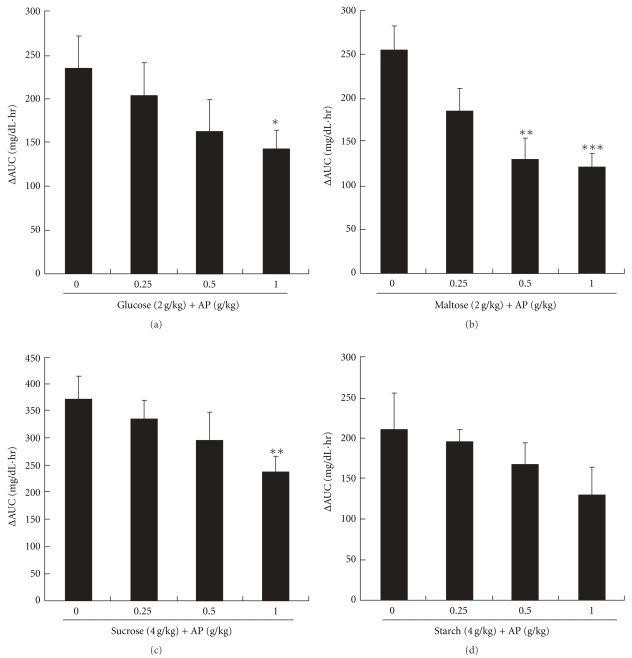
Carbohydrate tolerance tests in mice. ΔAUC values of blood glucose at 0 and 3 h in oral administration of AP (0–1.0 g kg^−1^) followed immediately by oral administration of glucose (2 g kg^−1^, (a), maltose (2 g kg^−1^, (b), sucrose (4 g kg^−1^, (c) or starch (4 g kg^−1^, (d) solution. ΔAUC is the difference of AUC and that in the no-carbohydrate group. Data represent means ± SD (*n* = 8). Dunnett's test: *P < .05, **P < .01 and ***P < .001 versus no-carbohydrate group.

Figure 4 shows the plasma glucose concentration profiles after oral administration of AP solution and glucose, maltose, sucrose or starch solution to mice.  Figure 5 depicts the plasma glucose ΔAUC up to 3 h post-administration. For glucose, maltose, or sucrose solution administered alone, the plasma glucose concentrations were highest at 30 min post-administration and decreased gradually. With administration of 1 g kg^−1^ AP, the rise in the plasma glucose concentrations after glucose and sucrose loading was significantly inhibited. In addition, the effect of AP on inhibiting the rise in glucose concentrations was greater after maltose loading than after glucose or sucrose loading. Administration of 1 g kg^−1^ AP resulted in a glucose ΔAUC of about 50% that for maltose alone. Furthermore, AP slightly inhibited the rise in plasma glucose concentrations after starch loading.

## 4. Discussion

Obesity is caused by excess caloric intake [16], and this can be improved by inhibiting pancreatic lipase activity and by inhibiting or delaying lipid absorption [17]. Inhibition of *α*-glucosidase activity and inhibition of carbohydrate absorption also play an important role in the prevention and treatment of diabetes [18]. In this study, we performed *in vitro* and *in vivo* experiments to evaluate the effects of AP on lipid and carbohydrates absorption.

AP significantly inhibited the rise in plasma triglyceride levels after olive oil administration in mice (Figure 3). This effect, based on results of the *in vitro* lipase activity inhibition test, is probably due to inhibition of lipase activity (Figure 1). The major constituents of AP are robinetinidol and fisetinidol, whose structures are similar to catechins [19–22]. Green tea polyphenols, which contain abundant catechins, at 0.5 mg ml^−1^ have also been reported to inhibit the activity of lipase to about 60% of control values [13]; this inhibitory effect is similar to that of AP observed in our study. In mice, oral administration of 1 g kg^−1^ apple polyphenols, which contain abundant proanthocyanidins, has been shown to inhibit lipid absorption, but apple polyphenols at 0.2 g kg^−1^ had no effect on lipid absorption [23]. In our study, oral administration of 0.25 g kg^−1^ AP to mice significantly inhibited lipid absorption, and at 0.5 g kg^−1^ AP, changes in plasma triglyceride levels were almost the same as those in normal controls (Figure 3). As green tea polyphenols [24, 25] and apple polyphenols [23, 26] have confirmed inhibitory effects on lipid absorption and obesity in humans, AP, which also inhibits lipid absorption, may have inhibitory effects against obesity in humans.

AP inhibited the rise in plasma glucose concentrations after glucose loading (Figures 4 and 5). In the small intestine, glucose is absorbed into the blood via a sodium-dependent glucose transporter (SGLT) and glucose transporter (GLUT) [27]. Polyphenols from acerola fruit have been reported to inhibit the rise in plasma glucose concentrations after glucose loading in mice; this may be attributable to inhibition of glucose uptake via SGLT and GLUT [8]. These findings suggest that AP contains constituents similar to polyphenols from acerola fruit and that these constituents, via inhibition of glucose uptake, inhibit the rise in plasma glucose concentrations after glucose loading. AP also significantly inhibited the rise in plasma glucose concentrations after maltose and sucrose loading in mice (Figures 4 and 5), and the inhibitory effects were more potent after maltose than sucrose loading. This finding corroborates the observation that maltase inhibitory activity was almost 3-fold higher than that of sucrase inhibitory activity (Figure 2). Catechins and catechin gallates, which are present in green tea, have been reported to have 2- to 4-fold greater inhibitory activity against maltase than against sucrase [14], suggesting that AP contains constituents with similar effects. AP also slightly inhibited the rise in plasma glucose concentrations after starch loading; this effect was weaker than that after glucose, maltose and sucrose loading (Figures 4 and 5). Starch in meals is hydrolyzed ultimately to glucose by amylase and then absorbed into the body. Blocking amylase activity has been effective for the treatment of diabetes [28, 29]. Green tea polyphenols [30] and black tea polyphenols [31] strongly inhibit amylase activity, and polyphenols in chestnut astringent skin inhibit the rise in plasma glucose concentrations after starch loading [32]. Although the active constituents of these polyphenols have not been identified, AP probably contains constituents similar to these polyphenols.

In similar studies, doses of 5–20 mg kg^−1^ orlistat and acarbose have been shown to have very potent inhibitory effects on lipid absorption and on rises in plasma glucose, respectively [33–35]. These same inhibitory effects were found in our *in vitro* study with AP at 0.5–1.0 g kg^−1^ but were markedly weaker than those of orlistat and acarbose. Incidentally, orlistat, which inhibits lipid absorption, is known to have side effects like steatorrhea for long-term administration in many patients [17]. In contrast, long-term administration (7 weeks) of AP produced no stool abnormalities (data not shown), most likely due to the milder lipase activity inhibition of AP than orlistat. Therefore, even long-term clinical administration of AP is less likely to cause side effects.

In recent years, polyphenols have been reported to possess antioxidant-like actions [36, 37]. Also, many of the natural extracts with antioxidant-like actions showed the non-specific inhibition toward lipase and/or glucosidase activities [8, 13, 23, 38]. AP inhibited not only lipase activities, but also maltase and sucrase activities. AP has also been reported to have antioxidant activities [39, 40]. These findings indicate that AP inhibits these enzyme activities non-specifically.

**Figure 6:**
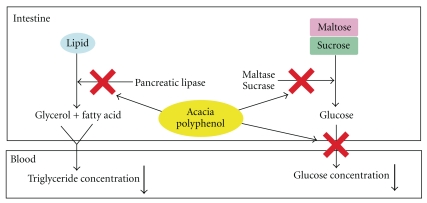
Hypothetical mechanisms of anti-obesity/diabetic actions of acacia polyphenol.

We previously reported that AP exerts anti-obesity and anti-diabetic effects by altering the expression of obesity- and diabetes-related genes in skeletal muscle, liver, and white adipose tissue [3]. In this study, AP showed inhibitory effects on lipase and glucosidase activities, as well as on the rise in plasma triglyceride and glucose concentrations after lipid and carbohydrate loading (Figure 6). These findings suggest that the anti-obesity and anti-diabetic effects of AP are attributable to not only to the absorbable constituents but also to the non-absorbable constituents that play roles in the gastrointestinal tract.
